# Ecological Responses to Extreme Flooding Events: A Case Study with a Reintroduced Bird

**DOI:** 10.1038/srep28595

**Published:** 2016-06-27

**Authors:** Andrea Soriano-Redondo, Stuart Bearhop, Ian R. Cleasby, Leigh Lock, Stephen C. Votier, Geoff M. Hilton

**Affiliations:** 1 Centre for Ecology and Conservation, College of Life and Environmental Sciences, University of Exeter, Cornwall Campus TR10 9EZ, UK; 2 Wildfowl & Wetlands Trust, Slimbridge, Gloucester, GL2 7BT, UK; 3 Royal Society for the Protection of Birds, The Lodge, Sandy, Bedfordshire, SG19 2DL, UK; 4 Environment and Sustainability Institute, University of Exeter, Cornwall Campus TR10 9EZ, UK

## Abstract

In recent years numerous studies have documented the effects of a changing climate on the world’s biodiversity. Although extreme weather events are predicted to increase in frequency and intensity and are challenging to organisms, there are few quantitative observations on the survival, behaviour and energy expenditure of animals during such events. We provide the first data on activity and energy expenditure of birds, Eurasian cranes *Grus grus*, during the winter of 2013–14, which saw the most severe floods in SW England in over 200 years. We fitted 23 cranes with telemetry devices and used remote sensing data to model flood dynamics during three consecutive winters (2012–2015). Our results show that during the acute phase of the 2013–14 floods, potential feeding areas decreased dramatically and cranes restricted their activity to a small partially unflooded area. They also increased energy expenditure (+15%) as they increased their foraging activity and reduced resting time. Survival did not decline in 2013–14, indicating that even though extreme climatic events strongly affected time-energy budgets, behavioural plasticity alleviated any potential impact on fitness. However under climate change scenarios such challenges may not be sustainable over longer periods and potentially could increase species vulnerability.

There is ample evidence that climate change has important consequences for biota and ecosystems^1^,^2^,^3^. A number of studies have shown that ecological and evolutionary responses to global warming are widespread and range from genetically-based adaptations, to population-level effects and geographic range shifts, as well as ecosystem-level reorganisation^2^,^4^,^5^,^6^,^7^. Moreover, climatic change is predicted to have other consequences than simply shifting average values. In particular it is expected to result in an escalation of extreme weather events^8^,^9^. However, research into the impact of extreme weather on biota presents daunting challenges: such events are rare, brief and difficult to predict^10^. Nevertheless, there is some evidence indicating that these short-term episodes can have long-term ecological consequences and have the potential to completely transform ecosystems^11^,^12^. They can lead to drastic reductions in population size of some species^13^,^14^,^15^,^16^, and in some instances extinctions have been associated with extreme weather events^17^. For example, several populations of Edith’s Checkerspot butterfly (*Euphydryas editha*) disappeared after extreme fluctuations in precipitation in California, USA, and it is known that some animal populations experience increased mortality during extreme winter conditions^18^,^19^. But some outcomes are much more indirect, opaque and difficult to predict, such as the impact of a single flood event upon a desert rodent community in Arizona, USA. This caused differential species-specific mortality and reset long-term population trends, leading to a complete rearrangement of the community, resulting in the displacement of a native by an invasive species^20^.

These population and community changes are a consequence of the sum of individual responses to a variable environment^21^,^22^ and most research on the effects of extreme weather events thus far has focused on population-level responses, neglecting the mechanisms that might underlie these population dynamics^21^. There is evidence to suggest that different individuals exhibit different physiological, morphological and behavioural adjustments to climatic challenges^17^,^22^,^23^,^24^,^25^, and that individuals in better body condition are better able to cope than individuals that were struggling beforehand^26^. In such instances, understanding the causes and consequences of individual variation in response to extreme weather events is required to better predict downstream demographic and population level consequences under different scenarios.

In the UK, evidence suggests that winter cyclones are becoming more common and their intensity is increasing^27^. Additionally, there are strong indications that the distribution of daily precipitation is altering, becoming more intense in winter and less intense in summer^28^; and the frequency of daily heavy rain events has increased from a 1 in 125 day event in the 1960 s to 1 a in 85 day event in 2009^28^,^29^. Some models also suggest that one-hour precipitation extremes could increase by ca. 14% per degree of warming in most parts of Europe^30^. Thus, there is likely to be an increase in the frequency and intensity of extreme winter precipitation in Europe. This in turn will lead to an increase in flooding events, and how organisms might respond to these extremes remains unknown.

In this study, we present data on the response to an extreme flooding event by Eurasian cranes (*Grus grus*; hereafter cranes), which were reintroduced in SW England from 2010 to 2014. Cranes are terrestrial feeders that rely on unflooded areas or shallow waters when feeding. Thus, a reduction of available foraging area because of extreme floods has the potential to impact fitness^31^. We monitored this crane population over three consecutive winters (2012–13, 2013–14 and 2014–15) and deployed GPS and accelerometry devices on multiple individuals (see methods for further details) to estimate their distribution, behaviour and energy expenditure. We performed monthly surveys of the population to determine survival and reproductive success. We also gathered data from satellite images and water gauges in the region to estimate flooding extent and dynamics. During winter 2013–14, the south of England experienced exceptional winter rainfalls, which led to the most extensive floods since the 19^th^ Century^32^. Here we use these data to assess the impact that this extreme weather event had on the individuals of the population, relative to the previous and the following year, in order to gain a better understanding of the mechanisms by which organisms cope with these extraordinary events.

## Results

### Fieldwork outcome

To estimate the response of birds to winter floods we focused our research on the months between November and March for three consecutive winters (2012–2015). We obtained telemetry locations from: 12 birds for the first winter (2012–13); 9 birds for the second winter (2013–14); and 7 birds for the last winter (2014–15). Five of the birds provided data in both the first and the second winter. We also recovered acceleration data from 7 birds in the first winter; from 4 birds in the second; and from 5 birds in the third winter. In this case, 2 birds provided data in both the first and the second winter. We did not use acceleration data from the third winter as the tags had different characteristics, with higher sensitivity, producing acceleration patterns that were not comparable with data from previous winters.

### Survival and reproduction

Monthly resightings showed that during the first winter (2012–13) the survival was 98.1% (1 crane out of 52 died), in the second winter (2013–14), when the extreme flooding event occurred, survival was 94.1% (4 cranes out of 68 died) while in the third winter survival was 96.2% (3 cranes out of 77 died). These overwinter survival estimates are generally higher than rates (≈90%) reported in studies from other locations^33^. All birds in this study were young; released as juveniles in the summers of 2010–2014. Thus in 2012–13, birds were in their first to third years, in their first to fourth years during 2013–14, and in their first to fifth years during 2014–15. The analysis showed that there were no significant differences in survival rates among the three winters (χ^2^ = 1.2, df = 2, P = 0.55). We were not able to test the effects of floods on subsequent breeding success because of the small number of nesting attempts among a population of immature birds; in 2013 there were none; in 2014 there were two unsuccessful breeding attempts; and in 2015 three pairs nested in the area, but only two chicks from one pair fledged.

### Flooding estimates

**Figure 1:**
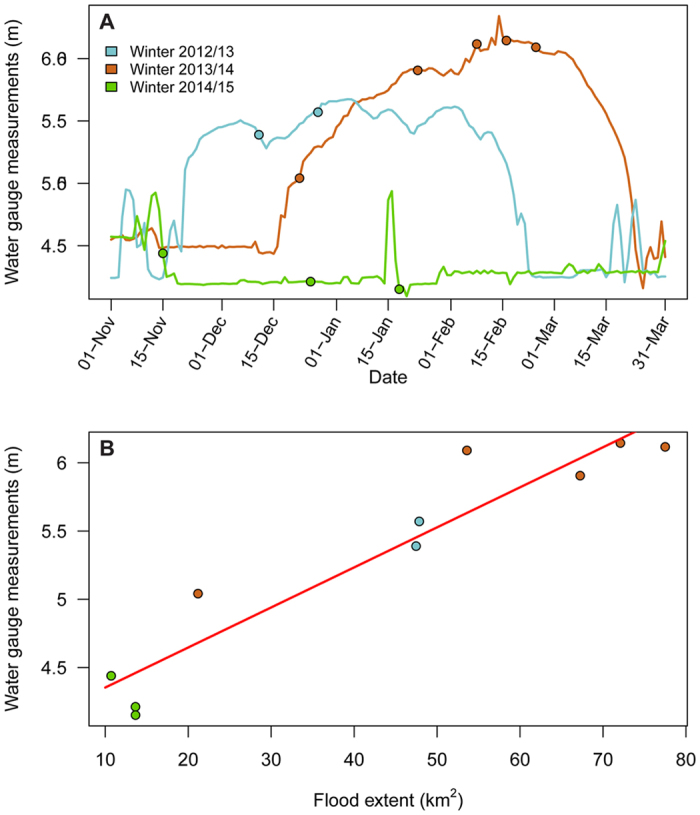
Panel (A): flood dynamics in the study site in the Somerset levels across the three winters. Points indicate dates with remote sensing data for the study area. Panel (B): relationship between water gauge measurements from the study area and the flood extent extracted from Landsat images, blue dots correspond to winter 2012–13, red dots to winter 2013–2014 and green dots to winter 2014–2015.

The extent of flooding in the Somerset Levels varied greatly across the three years of study, with the second winter having the most extensive floods (Fig. 1A). The flood dynamics also varied across years; in the first winter the peak of the flood was reached rapidly, in the second winter the flood started later and had a slower rate of increase until it reached the highest peak and in the third winter there was only a small and short flood (Fig. 1A). To quantify the flood extent, we downloaded 19 Landsat images spanning the duration of the study. Of these, 10 were cloud-free and met the necessary requirements for measuring the extent of flooding. We also obtained daily water gauge measurements from the study area to allow closer examination of flood progression. The correlation between flood extents estimated from the images and water gauge measurements was very strong and highly significant (linear regression, P < 0.001, R^2^ = 0.9, Fig. 1B). We then used this relationship to estimate daily flood extent from water gauge measurements across the three winters. These estimates revealed that, in the second winter, at the peak of the extreme flooding event, the area of the flood was 12% and 28% greater than at the highest point of the floods in the previous year and in the subsequent year, respectively.

### Bird distribution in response to flooding

**Figure 2:**
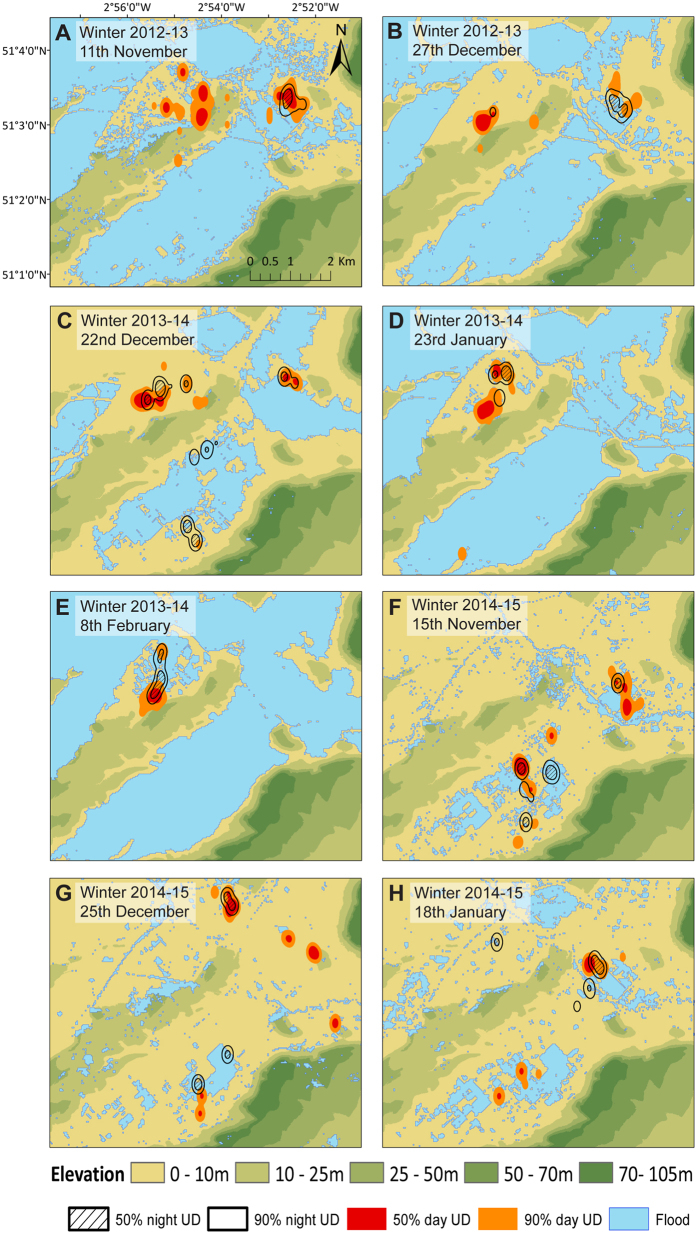
Crane active and roosting distribution as a function of flood extent for several dates across the three winters of study. Selected dates correspond to 9 out of 10 cloud-free satellite images that met the necessary requirements for measuring the extent of flooding. UD stands for utilization distribution. Map was created with ArcGIS version 10.2.2 (https://www.arcgis.com/).

**Figure 3:**
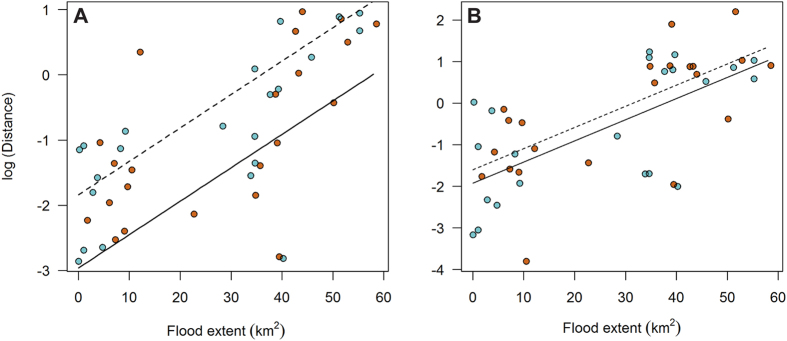
Fitted curves from the generalized least square model showing the relationship between flood extent and log-transformed distance between the used area and the preferred area for (**A**) active and (**B**) roosting periods by tracked cranes. We defined ‘preferred’ feeding areas as those which were used by cranes in winter 2014–15, when most of the area was unflooded. Black dots represent data from winter 2012–13, red dots represent data from winter 2013–14. Solid line represents the fitted curve in winter 2012–13 and the dashed line represents the fitted curve in winter 2013–14.

Crane distribution in the study area was linked to flood extent, and during the peak of the extreme flood cranes restricted their foraging activity to a small area that was partially unflooded (Fig. 2D,E). We defined ‘preferred’ feeding areas as those that were used by cranes in winter 2014–15, when most of the area was unflooded and their foraging habitat was much less restricted. We used this approach to account for seasonal variation in resource availability and to allow us to compare the same time points in each annual cycle. The distance between used areas and preferred areas was positively associated with flood extent (Table 1, Fig. 3A). The significant quadratic relationship between week of the season and distance from the preferred areas suggests that the difference in this distance among years decreased as the season progressed, but at a declining rate. In addition, there was evidence of temporal autocorrelation across birds in distance from preferred areas between weeks of the season within a given winter. However, when flood extent was accounted for, there was no difference in distance between winters and, based on model leave-one-out cross validation (LOO-CV) scores, winter was dropped from the best-fitting model. We found the same pattern for roost sites: the distance between used roost sites and preferred roost sites was positively associated with flood extent (Table 1, Fig. 3B). Cranes abandoned their usual roost areas and moved to partially flooded areas with shallow waters (Fig. 2). In contrast, distance between used and preferred roosting areas was not associated with week of the season and did not differ significantly between winters. Distance between used and the preferred roosting areas was autocorrelated across consecutive weeks of the season. In addition, there was less variation in this relationship during winter 2012–13 than in winter 2013–14.

### Bird energy expenditure and behaviour in response to flooding

**Figure 4:**
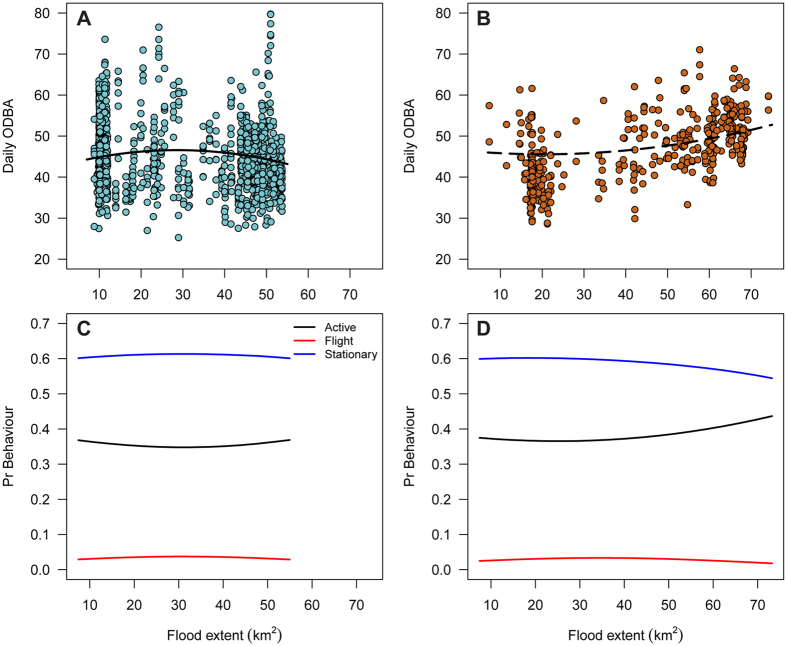
Left panels: relationship between flood extent and individual daily energy expenditure (summed daily overall dynamic body acceleration), for normal winter 2012–13 (**A**) and extreme flood winter 2013–14 (**C**). Fitted curves from multinomial models predicted only over the range of observed data in each year. Right panels: fitted curves from the multinomial model of behaviour in winter 2012–13 (**B**) and 2013–14 (**D**) showing the relationship between flood extent and the probability of performing one of three behaviours considered (active, flight or stationary).

To investigate how daily energy expenditure changed in relation to flooding we used the overall dynamic body acceleration (ODBA hereafter) as a proxy^34^,^35^. We found a significant quadratic relationship between summed daily ODBA and flood extent in both winters (Fig. 4A,B). However, the nature of this relationship differed significantly between winters, probably because of the different flood dynamics (Table 2). In winter 2012–13 the quadratic relationship was relatively flat but summed daily ODBA increased gradually with flood extension up to 30 km^2^ before gradually decreasing (Fig. 4A). In contrast, during winter 2013–14, when the extreme flood occurred, summed daily ODBA generally increased with flood extent, however the rate of increase accelerated as flood extent rose above 60 km^2^ (Fig. 4B). During the peak flood period ODBA was 15% higher than at low flood levels. Summed daily ODBA was also positively associated with Julian Date. In addition, there was evidence of temporal autocorrelation in daily ODBA across consecutive days. We also found evidence of significant among-individual and among-week variation in summed daily OBDA during the course of the study (Table 2).

The behaviour of birds was not significantly associated with flood extent during winter 2012–13, when water did not reach extreme levels (Table 3, Fig. 4C). In contrast, during winter 2013–14, when the extreme flood event occurred, there were significant quadratic relationships between flood extent and behaviour (Table 3). In particular, the probability of stationary behaviour decreased by 7% as flood extent increased whereas the probability of active behaviour increased by 8% at the peak of flood extent, which translates to around two extra hours of active behaviour each day (Fig. 4D). Stationary behaviour decreased as the winter progressed in both years. There was also significant among-individual variation in the probability of performing both stationary and flying behaviour in both years, suggesting that individual birds vary in the manner in which they respond to flooding events (Table 3).

## Discussion

Here we provide rare evidence of how closely monitored individual animals respond to an infrequent, catastrophic weather event of the type that appears to be increasing as our climate changes. Our study highlights the importance of combining population and individual responses to better understand how species respond to environmental fluctuations. We show that although the extreme weather event did not affect survival of birds, cranes abandoned favoured feeding and roosting areas, substantially increased feeding time and energy expenditure, and reduced resting time. Crane time- and energy-budget models suggest a non-linear response to flood extent, with the impact of floods only becoming apparent above a threshold that was exceeded in the extreme winter of 2013–14, but not in the more normal winters. This non-linear response is likely explained by the relationship between flood extent and the extent of available (unflooded) foraging area. During the two normal winters, multiple unflooded areas were available even at peak flood whereas during the peak of the extreme flood event there was a dramatic reduction of the potential feeding areas available to cranes.

There are two non-exclusive potential reasons behind the increase in foraging time. First, during the extreme flood cranes were feeding in suboptimal areas with low food density, which might have forced them to spend progressively more time searching for food to meet their energy demands^36^. Prolonged floods are known to have detrimental effects on soil macroinvertebrates, principally earthworms (a primary food of cranes), which can lead to a tenfold reduction in macroinvertebrate biomass in flooded areas compared with unflooded ones^37^. Furthermore, flooded areas remain poor during the following spring due to relatively slow re-colonization of soil invertebrates. Hence, our study population may have experienced a period of food shortage even after the main flood had receded and their preferred feeding areas were available^37^. Second, the social organisation of flocking cranes may also provide an explanation for overall increases in foraging time during the extreme event. Dominant cranes tend to displace subordinate birds from higher quality areas^38^. This in turn could generate increased searching in the displaced individuals and, indeed, we noted significant inter-individual differences in behaviours (Table 3).

Crane social structure also likely explains the observed individual variation in the baseline values of energy expenditure. This study was carried out on a reintroduced population, and we cannot rule out the possibility that they behave differently to birds from natural populations. However, we consider this to be unlikely; so far, the reintroduced population has not exhibited any abnormal or unexpected behaviour and has developed similar social structures as other crane populations. Crane social structure is a typical hierarchy, where dominants birds foraging in higher quality areas can achieve higher intake rates, spend less time searching and thus have lower energy expenditure than displaced individuals^38^. Under normal (i.e., not extremely flooded) conditions, subordinate cranes could benefit from remaining in lower foraging quality areas in order to avoid interference competition^38^, leading to a high ODBA variation among individuals. As outlined above, during the flood peak all cranes were forced to forage in a single area, which is likely to generate higher levels of interference which would explain why we observed an increase in energy expenditure across all individuals during this period. Nevertheless, the effects of the flood could have substantially differed among individuals, with subordinate and juvenile cranes being more susceptible to foraging restrictions.

Surprisingly, over-winter survival was extremely high during all three winters of study (~96%). The ability of individual cranes to adjust their time budgets (i.e., by increasing foraging effort and reducing resting time) seemed to be sufficient to buffer the effects of the flood. It is worth noting that during the extreme flood event temperatures were particularly mild, with average temperatures 2 °C higher than the previous year for the whole period (Horfield and Filton weather station, Bristol, UK). This could have reduced thermoregulatory costs and thus allowed cranes to better cope with the flood. The lack of direct fitness consequences is consistent with another recent study that revealed that behavioural flexibility in black-tailed godwits (*Limosa limosa*) mitigated the potential carry-over effects of record low temperatures^24^. Unfortunately, because of the small sample size we were not able to test the effects of floods on subsequent breeding success (cranes do not usually breed until 4 years of age and therefore most birds were reproductively immature both during the course of the extreme flood event and the subsequent breeding seasons). However, it has been documented that in heavily flooded areas, the sudden drop of water levels in spring can increase the risk of nest exposure to predators in some ground-nesting meadow birds^39^. Thus, the extreme flood could have had unexpected indirect effects on breeding success that we are unaware of.

Although short-term increases in energy expenditure had not apparent effects on immediate fitness in this instance, they have the potential to generate downstream consequences^40^. For example, increased energy expenditure during the breeding season has been associated with a twofold increase in mortality during the subsequent winter in kestrels (*Falco tinnunculus*)^40^. Likewise, the heavier workload imposed on barn swallows (*Hirundo rustica*) by tracking devices and the correspondent increase in energy expenditure reduced annual survival, delayed reproduction in the subsequent year and diminished clutch size^41^. In our case, the cumulative effect of short-term environmental stresses that will increase in frequency as a result of climate change are currently unclear, but they might influence the demography of populations facing such circumstances.

This study highlights the complexity of linking population responses with a rapidly changing environment. The flood conditions experienced in the Somerset Levels during the winter 2013–14, were sufficiently short – and were likely partially alleviated by mild temperatures – to allow individuals to cope with them through behavioural flexibility. However, not all individuals were able to respond in the same way, indicating that there may be limits to this flexibility and it seems reasonable to assume that more extreme conditions could generate fitness consequences. At the population level, responses are likely to be variable and idiosyncratic, with some species being more vulnerable than others. Moreover, under different climate change scenarios, where many organisms are already challenged by other factors^42^,^43^, the capacity to respond to extreme events may already be compromised and it could lead to increased species vulnerability^12^.

At this stage it is difficult to generalise the results obtained here on the impact of extreme flooding given the paucity of studies that address the topic. Moreover, since the opportunities to monitor those events are mostly subject to chance, and because the responses tend to be non-linear, such studies are unlikely to become commonplace. However, we must take advantage of these unusual opportunities to build up our understanding of the consequences these events will have on ecosystems.

## Methods

### Study site and fieldwork methods

The Somerset Levels and Moors (51° 2′ N, 2° 55′ W; 56,650 ha) are situated in SW England. The landscape is primarily composed of wetlands and natural or semi-improved grasslands grazed by livestock in low densities, and secondarily of arable fields of cereals and maize. Between 2010 and 2014, 94 captive-reared juvenile Eurasian cranes were released in the area. Before release, all individuals were marked with individual colour-ring combinations, and several individuals from each cohort were fitted with telemetry tags. Overall we deployed 8 leg-mounted solar-powered GPS-PTTs (North Star ST LLC); the total attachment mass was 71.7 g in 2010 and 63.7 g in 2012. We also deployed 31 GPS-UHF tags (e-obs GmbH), which were backpack mounted using an elastic harness; the mass of the tag plus harness was 68 g. In 2013 and 2014 we fitted 4 birds with solar-powered GPS-GSM tags (Ecotone Telemetry), leg-mounted on standard crane colour-rings; the overall mass was 51.5 g. The mass of the heavier devices (GPS-PTTs in 2010) only represented 1.3% of average cranes body mass (5400 g). To ensure that individuals could acclimate to the transmitter, the attachment was performed several days before the release and no side effects were observed after release in the study birds. Duty cycles differed among transmitters: PTT and GSM tags transmitted locations every 1–3 h, whereas GPS-UHF tags collected locations every 7 h. All location fixes from PTT tags were retrieved via the CLS tracking system (www.argos-system.org) and only standard class locations (3, 5) were retained. GPS-UHF tags recorded tri-axial body acceleration every 4 minutes in tags deployed in 2012 and 2013 and every 5 minutes in tags deployed in 2014. We did not include in the analysis the acceleration data from tags deployed in 2014, because, as explained in the results section, these tags had different characteristics and their sensitivity was higher so the acceleration patterns were not consistent with the previous tags. Bird handling and tagging protocols were carried out in accordance with relevant guidelines and regulations. These protocols were approved by the Wildfowl & Wetlands Trust Animal Welfare and Ethics Committee and the British Trust of Ornithology.

### Survival and reproduction

To estimate crane survival we estimated the proportion of birds that survived over winter (i.e known to be alive at start of the winter that were known to be alive at end of the winter). We searched and identified individually colour-ringed birds on a monthly basis for five consecutive months each winter, from the beginning of November to the end of March. This population is very localised, so the monthly surveys that have been carried out since the beginning of the reintroduction project in 2010, have high monthly resighting probabilities (ca. 94% monthly resighting probability), which obviates the need to control for re-sighting heterogeneity. Over the whole period no bird that has been unaccounted for more than five months has ever been resighted subsequently, therefore we considered a bird to be dead either when it was not recorded for more than 6 months or when its body was found. To test if there were significant differences in the survival rates among the three winters we used Test of Equal Proportions. We chose this approach because of the relatively invariant survival rates over the short duration of the study. Given the small sample size and the low number of individuals that died every winter it was not possible to include the age structure of the population in the analysis. We also performed surveys during three breeding season, from 2013 to 2015, to assess the effect of winter conditions on subsequent breeding season. However, cranes begin to establish pairs in their second or third year, with successful breeding occurring in the fourth or fifth year^44^. Thus, in 2013, the oldest birds in the population were only 3 years old and did not breed. This factor, combined with the small sample size from the following two breeding seasons, prevented us from performing statistical analysis to establish if floods had had an effect on breeding success.

### Flooding estimates

We generated flood maps from multi-temporal Landsat 7 and Landsat 8 Operational Land Imager (OLI) images between the winters of 2012–13 and 2014–15. The main limitation we faced was the low availability of cloud-free images and the number of images affected by the failure of the Scan Line Corrector^45^. Before any calculations were carried out, we converted Landsat 7 digital numbers (DN) into top of atmosphere (TOA) reflectance^46^. Similarly, Landsat 8 digital numbers were converted into TOA reflectance^47^. We used the Modified Normalized Difference Water Index (MNDWI), which uses one green band and one SWIR band to estimate the flooded surface^48^. The resulting maps were fed into the ISODATA algorithm in order to obtain a more detailed division of spectral classes. Subsequently, the classes corresponding with flooded surface were identified and selected. All GIS operations were carried out in QGIS 2.0 and ArcGIS 10.1.

To estimate the flood progression, we collected concurrent data from the water gauges in the study area. We used a generalized linear model to correlate the water gauge measurements with the flood extents extracted from the flood maps. Then we inferred flood extent from gauge measurements and obtained a dynamic model of flood progression over the three winters.

### Bird distribution in response to flooding

To characterize the daily (foraging) and nightly (roosting) distribution of cranes during the three consecutive winters, we used utilization distribution (UD) kernels (smoothing 0.001 and grid 2000). We estimated all kernels using *adehabitat* R package^49^. To assign each location to either active or roosting period we used the accelerometry data. We found a correlation between time of sunrise and the start of the active period (p < 0.0001) and between time of sunset and the start of the roosting period (p < 0.0001), so we were able to establish a threshold between the active period and the roosting period. The active period started ca. 63 minutes before sunrise and the roosting period started ca. 83 minutes after sunset.

To assess the impact of the flood extent on crane displacement from their preferred areas, we separately calculated the weekly 50% UD kernels for the active and the roosting period for the three winters. We then calculated the centroids of these kernels. Since the winter 2014–15 was particularly dry we used it as a reference to determine the preferred areas because the flood extent was relatively low and birds were free to use almost the whole area. We then calculated the minimum distance between the centroids for the winters 2012–13 and 2013–14 and the centroids of the preferred areas. We decided to use the minimum distance between the used areas and the preferred areas in order to focus on the displacement from the core areas and avoid the effect of exploratory behaviour far from the usual areas. To account for the possible variation in habitat among years we performed a complementary analysis, setting the sites used during the first week of November of winter 2012–13 and 2013–14 (instead of the whole winter 2014–15) as reference for preferred areas. We then calculated the minimum distance between those areas and the areas used in the subsequent weeks for the two winters. Analyses for both models were performed in the same manner. Results are qualitatively similar to those obtained in the previous model, thus only the first model is explained in the results section (Supplementary Table S1).

To examine whether the extent of flooding throughout the winter was linked to the distance between preferred sites (for active periods) we fitted a generalized least squares (GLS) model with log-transformed distance as our response variable in the R environment^50^ using the package nlme^51^. As fixed effect predictors in our model we included the weekly extent of flooding, the week in the winter, and winter (as a categorical variable). Prior to analysis, flood extent was standardized, to set the intercept to intermediate flood levels and ease model interpretation. Because both flood extent and week may have non-linear effects, we ran different models in which these continuous terms were included as simple linear effects, or as non-linear by fitting either a quadratic fixed effect or using b-splines. In addition, we included in our full model all two-way interactions between our fixed effects. Finally, we included an autocorrelation structure of order 1, using week as a continuous time covariate with year as the relevant grouping factor. This accounts for temporal autocorrelation between observations from adjacent weeks within a year. Model selection was performed using leave-one-out cross validation (LOO-CV) and selecting models with the lowest mean squared error (MSE) and predicted residual sum of squares (PRESS) scores^52^.

To model whether the extent of flooding had an effect on the distance between preferred roosting sites and used roosting sites, we used the same GLS modelling approach as described above, with log distance between preferred and used roosting sites as our response variable. However, due to evidence of heteroscedasticity in model residuals we also incorporated a variance function to allow for heterogeneity in residuals across the three years of our study^53^.

### Bird energy expenditure and behaviour in response to flooding

To investigate how daily energy expenditure changed in relation to flooding we used overall dynamic body acceleration (ODBA) as a proxy and calculated the summed ODBA of every tagged bird on a daily basis^34^,^35^. We used this measure as a response variable in a linear mixed effects model (LMM). As fixed effects in our model we included the daily flooding extent, the day of the year (measured as Julian date from the start of the appropriate year) and the winter in which records were taken. The continuous variables, daily extent of flooding and Julian date, were incorporated into models as linear effects or as non-linear effects by fitting either a quadratic curve or using b-splines. All two-way interactions between variables were also included in our full models before simplification took place. We included bird ID as a random effect to account for the fact that summed daily ODBA measures taken from the same individual may not be independent, and week of winter to account for the lack of independence of measures taken within the same week. In addition, we included a temporal autocorrelation structure of order 1 using Julian date as a continuous time covariate, and with year as the relevant grouping factor, to account for temporal autocorrelation in summed daily ODBA values. Due to evidence of heteroscedasticity in model residuals we also allowed the residual variance in our models to differ across years. Model selections was performed using K-fold cross validation where K = 5 in order to calculate MSE and identify the best fitting model. K-fold cross validation represents a variation on LOO-CV and was developed as an alternative to the computationally expensive LOO-CV^54^ because of the large number of data points in the ODBA models.

To elucidate bird behaviour through acceleration patterns, we used AcceleRater, a web application that provides supervised machine learning models that classify unknown behaviour through already labelled data^55^. We classified crane behaviour into three categories: stationary, active and flying. To do so, we filmed birds fitted with GPS-UHF tags. We obtained 118 instances of stationary behaviour, 310 of active behaviour and 89 of flying behaviour. We ran several different fitting techniques (K nearest neighbours, Linear support vector machines, Radial basis function kernel support vector machines, Decision tree, Random forest and Naïve Bayes) using these data. The results were cross validated with the Train-Test split method to assess the recall, accuracy and precision of the output of a given model. We selected the Random Forest method to classify the unlabelled data since it obtained the highest scores in recall, precision and accuracy, 95.8%, 96.0% and 97.4% respectively. Each crane behaviour-type was classified in this way every 15 minutes, generating a vector of behavioural responses Yi that take one of *J* = 3 discrete values. With such categorical data the multinomial distribution can be used to estimate the probability that the *i*th response falls into the *j*th category (equation 1).


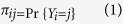


Where, for example, πi1 is the probability that *i*th response is classed as ‘active’. Moreover, because our response categories are mutually exclusive and exhaustive we have:


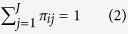


That is, the probabilities sum to one for each response and we have J-1 parameters because once we know the probability of Y_i_ being classed as active and of being classed as flying then by simple subtraction we know the probability that Y_i_ will be classed as stationary (equation 2). To model the behaviour of cranes we used a Bayesian multinomial mixed-effects model within the MCMCglmm R package^56^, with three response variables, active, flying and stationary. As fixed effects within our model we included Julian date, winter and daily flood extent. Visual inspection of data suggested the inclusion of a quadratic flood extent effect and an interaction between flood extent and winter. Initially, we incorporated a two-way interaction between flood extent and winter in our multinomial models. However, once the existence of a two-way interaction between winter and flood extent was confirmed (95% CRI of interaction coefficient did not overlap 0) we ran separate multinomial models for each winter of our dataset for ease of subsequent interpretation. By doing so, we could still capture the interaction as we were still modelling separate flood extent coefficients for each year. As random effects in our models we included bird ID and Julian date to account for the potential lack of independence on measurements taken on the same bird or the same day. We used non-informative priors in our multinomial models and ran 3 MCMC chains for 150,000 iterations, with a burn-in of 30,000 and a thinning interval of 10^56^. Convergence of chains was assessed using the Gelman-Rubin diagnostic and model fit was assessed using a posterior predictive check^57^.

As crane behaviour was assessed (via accelerometry) every 15 minutes, consecutive behaviours in time were likely to be highly correlated; we thus added a lagged dependent variable (the behaviour previously performed by the bird) to the original model to reduce the occurrence of autocorrelation. Results are similar to those obtained in the original model and our conclusions are thus unaffected by potential behavioural autocorrelation (Supplementary Table S2).

## Additional Information

**How to cite this article**: Soriano-Redondo, A. *et al*. Ecological Responses to Extreme Flooding Events: A Case Study with a Reintroduced Bird. *Sci. Rep.* **6**, 28595; doi: 10.1038/srep28595 (2016).

## Supplementary Material

Supplementary Information

## Figures and Tables

**Table 1:** Generalized least square model of log-transformed distance between the used area and the preferred area during active and roosting periods.

Period	Variable	Coefficient	Lower 95% CI	Upper 95% CI	P-value
Active	Intercept	0.11	−1.26	1.47	0.87
	Flood Extent	0.99	0.42	1.56	<0.001
	Week	−0.34	−0.67	−0.010	0.045
	Week^2^	0.014	−0.0015	0.031	0.075
	Winter	−0.017	−1.88	1.85	0.95
	Corr. struct: Week/Winter	0.37	0.10	0.74	NA
Roosting	Intercept	−0.18	−1.24	0.88	0.73
	Flood Extent	0.98	0.62	0.98	<0.001
	Week	−0.035	−0.096	0.025	0.24
	Winter	0.32	−0.62	1.27	0.49
	Corr. struct: Week/Winter	0.31	−0.075	0.61	NA
	Var. function: 2^nd^ Winter	0.52	0.33	0.81	NA

We defined ‘preferred’ feeding areas as those which were used by cranes in winter 2014–15, when most of the area was unflooded. Note that a p value cannot be calculated for the temporal autocorrelation structure (Corr. struct). We used leave-one-out cross validation (LOO-CV) scores to select the best model, including as predictors the weekly extent of flooding, the week in the winter, and winter. In the active period, based on model LOO-CV scores, the fixed effect was not included in our best fitting model but its coefficient is reported here for completeness. In the roosting period, the variance function allows within-group variance to differ between years. In this case the reported coefficient for the second winter represents the ratio between the standard deviation in the second winter relative to that in the first winter. Based on model LOO-CV scores week and winter were not included in the best model but coefficients are presented here for completeness. N = 44 observations.

**Table 2:** Linear mixed-effects model of daily ODBA.

Variable	Coefficient	Lower 95% CI	Upper 95% CI	P- value
Intercept	46.02	43.63	48.41	<0.001
Flood Extent	−1.27	−2.28	−0.27	0.0134
Flood Extent^2^	−1.47	−2.61	−0.35	0.0104
Julian Date	3.59	3.062	4.13	<0.001
Winter	−1.34	−6.041	3.42	0.53
Winter × Flood Extent	2.41	0.99	3.82	<0.001
Winter × Flood Extent^2^	2.48	0.91	4.061	0.002
Corr. struct: J. Date/Winter	0.057	0.018	0.18	NA
Var. function: 2^nd^ Winter	1.19	1.10	1.29	NA
Bird ID Random Effect	σ = 2.89	1.74	4.82	NA
Week ID Random Effect	σ = 2.49	2.011	3.091	NA

Random effects here represent among individual standard deviation in intercepts. Note that a p value cannot be calculated for the temporal autocorrelation structure (Corr. struct). Model selections was performed using K-fold cross validation where K = 5, including as predictors the daily extent of flooding, the Julian date in the winter, and winter. As random effects in the model we included bird ID and week. N = 1469 observations taken across two years. Number of weeks = 44. Number of birds = 11.

**Table 3:** Coefficients for Bayesian multinomial model of crane behavioural categories based on accelerometry data.

Winter	Variable	Coefficient	Lower 95% CRI	Upper 95% CRI
2012–13	Flying	**−2**.**48**	**−3**.**24**	**−1**.**74**
	Stationary	0.62	−0.034	1.27
	Flying × Julian Date	0.033	−0.045	0.11
	Stationary × Julian Date	**−0**.**12**	**−0**.**16**	**−0**.**077**
	Flying × Flood Extent	−0.13	−0.26	0.010
	Stationary × Flood Extent	−0.031	−0.10	0.041
	Flying × Flood Extent^2^	**−0**.**20**	**−0**.**37**	**−0**.**031**
	Stationary × Flood Extent^2^	−0.051	−013	0.039
	Bird ID × Flying Random Effect	σ = 0.86	0.54	1.70
	Bird ID × Stationary Random Effect	σ = 0.76	0.47	1.48
2013–14	Flying	**−3**.**29**	**−4**.**36**	**−2**.**16**
	Stationary	0.29	**−**0.73	1.29
	Flying × Julian Date	**−**0.036	**−**0.17	0.096
	Stationary × Julian Date	**−0**.**18**	**−0**.**24**	**−0**.**13**
	Flying × Flood Extent	**−0**.**20**	**−0**.**34**	**−0**.**065**
	Stationary × Flood Extent	**−0**.**065**	**−0**.**12**	**−0**.**011**
	Flying × Flood Extent^2^	0.012	**−**0.14	0.18
	Stationary × Flood Extent^2^	**−0**.**053**	**−0**.**096**	**−0**.**011**
	Bird ID × Flying Random Effect	σ = 0.85	0.47	2.22
	Bird ID × Stationary Random Effect	σ = 0.81	0.44	2.083

Coefficients show the effect of predictors on the probability of performing stationary and flying behaviour respectively compared to active behaviour. Random effects represent among-individual standard deviation in the probability of performing flying and stationary behaviour respectively. Winter 2012–13: N = 126,840 observations from 7 birds. Winter 2013–14: N = 49,440 observations from 4 birds. Fixed effects where 95% CRI does not cross zero are highlighted in bold.
