# Supplementary material for: Ecological Responses to Extreme Flooding Events: A Case Study with a Reintroduced Bird
Source: Sci Rep. 2016 Jun 27;6:28595. doi: 10.1038/srep28595 (PMC4922006; doi:10.1038/srep28595)
Supplement: Supplementary Information [file srep28595-s1.pdf]

Supporting Information

Ecological responses to extreme flooding events: a case study with a reintroduced bird

Andrea Soriano-Redondo*[1,2,3], Stuart Bearhop[1], Ian R. Cleasby[1], Leigh Lock[3], Steve Votier[4], Geoff M. Hilton[2]

[1]Centre for Ecology and Conservation, College of Life and Environmental Sciences, University of Exeter, Cornwall Campus TR10 9EZ, UK.

[2]Wildfowl & Wetlands Trust, Slimbridge, Gloucester, GL2 7BT, UK.

[3]Royal Society for the Protection of Birds, The Lodge, Sandy, Bedfordshire, SG19 2DL, UK

[4]Environment and Sustainability Institute, University of Exeter, Cornwall Campus TR10 9EZ, UK.

*as746@exeter.ac.uk

Supplementary Table S1. Generalized least square model of log-transformed distance between used and preferred areas.

Period	Variable	Coefficient	Lower 95% CI	Upper 95% CI	P-value
Active	Intercept	-2.55	-3.43	-1.66	< 0.001
	Flood Extent	0.029	0.011	0.046	0.0018
	Flood Extent ^{2}	-0.001	-0.003	-0.0001	0.032
	Week	0.13	0.068	0.18	< 0.001
	Winter	0.76	-0.002	1.53	0.051
	Corr. struct: Week/Winter	0.36	0.091	0.76	NA
Roosting	Intercept	-2.28	-3.78	-0.77	0.004
	Flood Extent	0.041	0.068	0.10	< 0.001
	Week	0.058	-0.008	0.18	0.07
	Winter	0.83	-0.49	2.16	0.21
	Corr. struct: Week/Winter	0.51	0.21	0.80	NA
	Var. function: 2nd Winter	0.68	0.44	1.06	NA

Supplementary Table S1. Generalized least square model of log-transformed distance between used areas: (1) during the first week of November of 2012 and the remaining weeks of the winter 2012-2013 and (2) during the first week of November of 2013 and the remaining weeks of the winter 2013-2014, for the active and roosting periods. We chose the first week of November of each year as a reference for preferred area because most of the area was unflooded and birds were free to use

the whole study area. Note that a p value cannot be calculated for the temporal autocorrelation structure (Corr. struct). We used leave one out cross validation (LOO-CV) scores to select the best model, including as predictors the weekly extent of flooding, the week in the winter, and winter. In the roosting period, the variance function allows within-group variance to differ between years. In this case the reported coefficient for the second winter represents the ratio between the standard deviation in the second winter relative to that in first winter. Based on model LOO-CV scores winter was not included in the best model but the coefficient is presented here for completeness. N = 42 observations.

Supplementary Table S2. Coefficients for Bayesian multinomial model of crane behavioural categories based on accelerometry data.

	Variable	Coefficient	Lower 95% CRI	Upper 95% CRI
2012-13	Flying	**-2.73**	**-3.44**	**-2.03**
	Stationary	**-0.97**	**-1.47**	**-0.41**
	Flying × Julian Date	0.041	-0.033	0.11
	Stationary × Julian Date	**-0.071**	**-0.11**	**-0.042**
	Flying × Flood Extent	-0.12	-0.25	0.010
	Stationary × Flood Extent	-0.022	-0.083	0.003
	Flying × Flood Extent^2	**-0.19**	**-0.35**	**-0.036**
	Stationary × Flood Extent^2	-0.037	-011	0.039
	Flying × Flying $_{t-1}$	**0.74**	**0.62**	**0.86**
	Stationary × Flying $_{t-1}$	**0.18**	**0.091**	**0.26**
	Flying × Stationary $_{t-1}$	**0.50**	**0.43**	**0.58**
	Stationary × Stationary $_{t-1}$	**2.81**	**2.77**	**2.84**
	Random Effects			
	Bird ID × Flying	σ = 0.83	0.53	1.62
	Bird ID × Stationary	σ = 0.66	0.41	1.26
2013-14	Flying	**-3.45**	**-4.51**	**-2.37**
	Stationary	**-1.31**	**-2.30**	**-0.28**
	Flying × Julian Date	-0.0021	-0.15	0.11
	Stationary × Julian Date	**-0.11**	**-0.16**	**-0.071**
	Flying × Flood Extent	**-0.19**	**-0.33**	**-0.065**
	Stationary × Flood Extent	**-0.057**	**-0.10**	**-0.005**
	Flying × Flood Extent^2	0.051	-0.15	0.17
	Stationary × Flood Extent^2	**-0.043**	**-0.078**	**-0.006**
	Flying × Flying $_{t-1}$	**0.72**	**0.44**	**0.99**
	Stationary × Flying $_{t-1}$	**0.26**	**0.10**	**0.43**
	Flying × Stationary $_{t-1}$	**0.51**	**0.37**	**0.65**
	Stationary × Stationary $_{t-1}$	**3.02**	**2.97**	**3.07**
	Random Effects			
	Bird ID × Flying	σ = 0.83	0.45	2.18
	Bird ID × Stationary	σ =0.79	0.43	2.08

Supplementary Table S2. Coefficients for Bayesian multinomial model of crane behavioural categories based on accelerometry data. Coefficients show the effect of predictors on the probability of performing Stationary and Flying behaviour respectively. Random effects represent among-individual standard deviation in the probability of performing Flying and Stationary behaviour

respectively. To account for the existence of autocorrelation in the model we included lagged dependent variables (t-1). Winter 2012-13: N = 126,833 observations from 7 Birds. Winter 2013-14: N = 49,436 observations from 4 Birds. Fixed effects where 95% CRI does not cross zero highlighted in bold.
